# Autoimmunity and autoinflammation: A systems view on signaling pathway dysregulation profiles

**DOI:** 10.1371/journal.pone.0187572

**Published:** 2017-11-03

**Authors:** Arsen Arakelyan, Lilit Nersisyan, David Poghosyan, Lusine Khondkaryan, Anna Hakobyan, Henry Löffler-Wirth, Evie Melanitou, Hans Binder

**Affiliations:** 1 Bioinformatics Group, Institute of Molecular Biology, National Academy of Sciences RA, Yerevan, Armenia; 2 Department of Bioinformatics and Bioengineering, Russian-Armenian University, Yerevan, Armenia; 3 Zaven and Sonia Akian College of Science and Engineering, American University of Armenia, Yerevan, Armenia; 4 Group of Immune Response Regulation, Institute of Molecular Biology, National Academy of Sciences RA, Yerevan, Armenia; 5 Interdisciplinary Centre for Bioinformatics, University of Leipzig, Leipzig, Germany; 6 Department of Parasitology and Insect Vectors, Institut Pasteur, Paris, France; College of Bioinformatics Science and Technology, CHINA

## Abstract

**Introduction:**

Autoinflammatory and autoimmune disorders are characterized by aberrant changes in innate and adaptive immunity that may lead from an initial inflammatory state to an organ specific damage. These disorders possess heterogeneity in terms of affected organs and clinical phenotypes. However, despite the differences in etiology and phenotypic variations, they share genetic associations, treatment responses and clinical manifestations. The mechanisms involved in their initiation and development remain poorly understood, however the existence of some clear similarities between autoimmune and autoinflammatory disorders indicates variable degrees of interaction between immune-related mechanisms.

**Methods:**

Our study aims at contributing to a holistic, pathway-centered view on the inflammatory condition of autoimmune and autoinflammatory diseases. We have evaluated similarities and specificities of pathway activity changes in twelve autoimmune and autoinflammatory disorders by performing meta-analysis of publicly available gene expression datasets generated from peripheral blood mononuclear cells, using a bioinformatics pipeline that integrates Self Organizing Maps and Pathway Signal Flow algorithms along with KEGG pathway topologies.

**Results and conclusions:**

The results reveal that clinically divergent disease groups share common pathway perturbation profiles. We identified pathways, similarly perturbed in all the studied diseases, such as PI3K-Akt, Toll-like receptor, and NF-kappa B signaling, that serve as integrators of signals guiding immune cell polarization, migration, growth, survival and differentiation. Further, two clusters of diseases were identified based on specifically dysregulated pathways: one gathering mostly autoimmune and the other mainly autoinflammatory diseases. Cluster separation was driven not only by apparent involvement of pathways implicated in adaptive immunity in one case, and inflammation in the other, but also by processes not explicitly related to immune response, but rather representing various events related to the formation of specific pathophysiological environment. Thus, our data suggest that while all of the studied diseases are affected by activation of common inflammatory processes, disease-specific variations in their relative balance are also identified.

## Introduction

Epidemiological studies provide increasing evidence for the rise in prevalence of immune-related disorders including autoimmune and allergic diseases in Western countries [1–3]. It is predicted that the incidence of chronic inflammatory disorders, particularly autoimmune diseases, such as type 1 diabetes, Crohn’s disease, rheumatoid arthritis and multiple sclerosis, will grow even more rapidly during the next several decades [1,4,5]. Moreover, chronic inflammation is being recognized as an important trigger and contributor to the development and progression of various other human complex diseases, such as certain cancers, atherosclerosis, strokes and ischemic heart diseases, and even psychiatric disorders (schizophrenia and post-traumatic stress disorder) [6–10].Therefore, understanding the molecular mechanisms underlying the development of inflammatory disorders will have significant impact on public health.

Autoimmune diseases(AI) are characterized by dysfunction of the immune system leading to loss of immune tolerance against self-tissues, by the presence of autoreactive T and B cells, and by a complex pathogenesis of multifactorial etiology, whereas genetics and environmental factors together are responsible for disease onset [11,12].There are more than 80 such conditions affecting susceptible human subjects [13]. Autoimmune conditions may be systemic (targeting multiple organs and tissues), as is the case of lupus, or tissue specific, as is the case of multiple sclerosis (against myelin) or type 1 diabetes (against pancreatic beta cells). The simultaneous presence of several autoimmune diseases is observed in some cases, pointing at the possibility of a shared origin and/or mechanisms [14].

Autoinflammatory diseases (AIF) are a relatively new and expanding group of self-directed inflammatory disorders, clinically described as periodic fever syndromes but also with episodes of acute inexplicable inflammation involving the innate immune system [15,16]. They are characterized by inflammatory episodes at disease-prone sites, in the absence of autoreactive T cells and high autoantibody titers [17,18]. Despite the differences in primary players, they share common characteristics with AI diseases, such as self-tissue directed inflammation in the absence of an obvious infectious trigger or injury. While in AIFs the innate immune system directly causes tissue inflammation, in AIs the innate immune system activates the adaptive system and this later activates the inflammatory process [15]. The autoinflammatory syndromes include a subset of hereditary conditions characterized by recurrent episodes of fever and self-resolving attacks of systemic inflammation without microbial infection or autoimmunity. There are several AIF associated with genetic mutations affecting the innate immune system, the primary defense system against foreign antigens [19]. Such genetic mutations have been identified affecting genes encoding for the tumor necrosis factor receptor (TNFR1) as is the case for the autosomal dominant TNF receptor associated syndrome (TRAPS) [20] or the gene encoding for mevalonate kinase (MVK) responsible for the HIDS syndrome. Mutations of the *MVK* gene are responsible for an increase of mevalonic acid concentrations, elevated levels of IgD in the serum and also an increased secretion of IL-1β [21,22]. Similarly several autoinflammatory syndromes are related with the gene encoding for pyrin [22]. The PAPA (pyogenic arthritis, pyoderma gangrenosum and acne) syndrome, a dominantly inherited autoinflammatory condition is associated with mutations in the gene encoding for proline serine threonine phosphatase-interacting protein (PSTPIP1) that interacts with pyrin [23]. Pyrin and PSTPIP1 proteins are associated with the cytoskeleton in myeloid/monocytes and their interaction contributes to increased IL-1β production, NFkB activation and apoptosis [24]. Finally the connection between pyrin and autoinflammation is observed in the Cryopyrin-Associated Periodic Syndromes (CAPS) in which mutations of genes encoding for the components of the proteins involved in the inflammasome (*NLRP3*) are implicated [25–27].The phenotypic heterogeneity characteristic of AI and AIF diseases, does not necessarily reflect fundamental genetic or mechanistic differences between these groups. Indeed, while some gene variants and SNPs are specific to a particular disease [11,28], others predispose an individual to the development of multiple disorders, which indicates that shared mutations may affect genes or pathways implicated in the pathogeneses of several diseases [29,30]. Moreover, while the mechanisms and causes of development may differ in various diseases, the downstream effects occurring after the disease onset may be similar. This similarity can potentially underlie responsiveness of different types of diseases to the same type of treatment (e.g. glucocorticoids) [31–33] indicating the presence of shared drug targets. However, the global picture of molecular mechanisms underlying the similarities and specificities of chronic inflammatory disorders is not completely understood and is addressed in this study, using gene expression data to compare activation patterns of selected pathways in a subset of AI and AIF diseases.

Luckily, the advances in high-throughput biological data measurements, explosive growth of data in various public repositories and development of new bioinformatics algorithms for data analysis, already provide an opportunity to address the above mentioned issues from a systems biology viewpoint. Previously, we had developed a bioinformatics pipeline for pathway perturbation based analysis and similarity/specificity assessment of disease groups, and have applied it to a number of lung diseases [34]. The obtained results had confirmed the validity of our methodology, as well as had led us to draw new conclusions on pathological characteristics of lung diseases. In the present work, we have used our approach for global assessment of similarities and specificities of downstream molecular events of chronic inflammation through evaluation of pathway activity changes in autoimmune and autoinflammatory disorders.

We have combined several publicly available datasets for autoimmune and autoinflammatory diseases in an attempt to uncover their common and specific pathobiological features. Our goal was to include as many conditions as possible, while in the meantime minimizing heterogeneity of microarray platforms and source tissue used for transcriptome measurement. These criteria left us with four monogenic autoinflammatory, four polygenic autoinflammatory disorders sharing characteristics with autoinflammation and autoimmunity, and four polygenic autoimmune conditions (Table 1). Monogenic autoimmune disorders were not included in our study, as to our knowledge, respective transcriptome data meeting our selection criteria were not available. Even though the apparent heterogeneity of the disorders selected in our study presented a challenge for identification of common mechanisms, the existing similarities and, in particular, the common inflammatory component of these disorders, remained intriguing and encouraged us to undertake a comparative gene expression analysis of a large set of existing transcriptome data.

**Table 1:** Description of the samples and datasets included in the study.

Disease	Type*	Abbrev.	GEO Acc.	Number of Cases	Number of Controls	Array Platform
Type 1 Diabetes	AI	T1D	GSE55100	12	10	Affymetrix Human Genome U133 Plus 2.0 Array
Multiple sclerosis	AI	MS	GSE21942	12	15	Affymetrix Human Genome U133 Plus 2.0 Array
Systemic lupus erythematosus	AI	SLE	GSE50772	61	20	Affymetrix Human Genome U133 Plus 2.0 Array
Sjögren's syndrome	AI	SS	GSE48378	11	16	Affymetrix Human Exon 1.0 ST Array
Cryopyrin associated periodic syndrome	AIF	CAPS	GSE43553	23	20	Affymetrix Human Genome U133A 2.0 Array
Hyper IgD Syndrome (HIDS) (mutations in MVK gene)	AIF	MVK	GSE43553	8	20	Affymetrix Human Genome U133A 2.0 Array
PAPA Syndrome (mutations in PSTPIP1 gene)	AIF	PSTPIP1	GSE43553	6	20	Affymetrix Human Genome U133A 2.0 Array
TNF receptor associated periodic syndrome	AIF	TRAPS	GSE43553	29	20	Affymetrix Human Genome U133A 2.0 Array
Behcet’s disease	PAIF	BD	GSE17114	15	14	Affymetrix Human Genome U133 Plus 2.0 Array
Crohn’s disease	PAIF	CD	GSE3365	59	42	AffymetrixHuman Genome U133A Array
Ulcerative colitis	PAIF	UC	GSE3365	26	42	Affymetrix Human Genome U133A Array
Juvenile Idiopathic Arthritis	PAIF	JIA	GSE67596	22	15	Affymetrix Human Genome U133 Plus 2.0 Array

* AI—autoimmunity, AIF—monogenic autoinflammation, PAIF—polygenic autoinflammation (20, 21)

Our study offers a systems biology approach for comparative analysis of gene expression data from cohorts carrying apparent divergent diseases with common immune related manifestations. Despite the limitations discussed below, such studies may contribute to identification of common pathways in autoimmunity and autoinflammation, and have the potential to facilitate development of new experimental strategies for understanding the molecular mechanisms underlying the interplay between those processes.

## Material and methods

### Data sources

We have used data deposited at Gene Expression Omnibus (GEO), which is the largest repository for microarray gene expression studies [35]. The search was performed with keywords “autoinflammation”, “autoimmunity” and limited to human samples. We were aimed at selection of the most similar study designs (sample source tissue type, availability of controls) and microarray platforms. Thus, only datasets derived from peripheral blood mononuclear cells (PBMCs), as the most frequent sample source; and Affymetrix platforms, as the most prevalent ones, were chosen.

Our selection resulted in eight microarray datasets containing gene expression profiles of PBMC samples from patients with autoimmune and (or) autoinflammatory diseases, as well as healthy subjects. All of the platforms were Affymetrix microarrays, of which five were Human Genome U133 Plus 2.0, four were Human Genome U133A 2.0, two were Human Genome 133A, while only one was from Human Exome 1.0 ST Array platforms (Table 1). Data on Sjögren's Syndrome was the only dataset in our analysis that was not derived from Human Genome U133 series platforms, since there were no alternative datasets for this condition in GEO at the time of conducting this study. The effect of possible systematic biases introduced by biological and technical variation in our dataset collection is addressed below.

Only untreated samples were included in the analyses. Dataset accessions, descriptions and sample counts are presented in Table 1. For detailed information on subject characteristics, disease stage, sample preparation and scan protocol and low level data processing, the reader is referred to the GEO database pages under sample accessions provided in Table 1.

### Dataset preprocessing

Raw Affymetrix CEL files have been downloaded for all the datasets, except for GSE3365 (gene expression in Crohn’s disease and Ulcerative colitis), for which no raw data were available. Probe signal intensity conversions, RMA normalization and chip annotation were performed using the “affy” package for R [36]. The GSE48378 exon array dataset (gene expression in Sjögren's syndrome) was preprocessed using the “oligo” package [37], since “affy” is not intended for analysis of Affymetrix Human Exon arrays. The GSE3365 dataset was annotated based on platform information provided by GEO (GPL96: Affymetrix Human Genome U133A Array), and preprocessed using Affymetrix MAS5 algorithm.

All the genes with known Entrez IDs were annotated, and expression values for multiple probes of the same gene were averaged. The mean gene expression values of the controls within each dataset were used as reference to calculate the log fold changes (logFC) for each gene. The logFC values were then anti-logged to linear scale FC values.

### Pathway signal flow calculation

Pathway signal flow analysis was performed as described previously [38]. Briefly, 168 signaling and metabolic pathway maps were obtained from the KEGG pathways database, with the exclusion of disease and drug response pathways. The KEGG pathways are represented as graphs, where nodes are gene groups with similar functions and edges are interactions between them. The interactions are classified into “activation” and “inhibition” types. Each pathway has more than one input node, and more than one output (sink) node. The network of interactions that end with a single sink node is called a pathway branch. Note that each pathway may have many branches ending in specific sink nodes, where each may be associated with a different biological event or outcome. Thus, it is reasonable to evaluate activities of pathway branches individually [38].

The Pathway Signal Flow (PSF) algorithm for calculation of the activity values of pathway branches is described in detail in [39–41]. In short, PSF first assigns gene expression fold change (FC) values of the member genes to pathway nodes, and then sequentially updates the target node values for each source-target interaction: starting from the input nodes and finishing at the sinks. The target node values are updated depending on the type of interaction: ‘activation’ means that the greater the FC value at the source is, the greater the FC value at the target will be; whilst ‘inhibition’ has the opposite effect. In the end, the PSF algorithm returns the PSF values of the sink nodes for each pathway branch. The PSF values estimate the pathway activity fold change compared to the controls. Thus, values less than 1, are indicative for de-activation, while values greater than one for activation of the respective pathway branch.

We have evaluated in total 1825 sinks for the 168 pathways analyzed. Thus, each dataset is described with a vector of 1825 PSF values. Each sink is described by a profile of PSF values across all samples.

To estimate the possible systematic effect of biological and technical variation, we have substantially extended our analyses based on additional datasets (S1 File). We have demonstrated that there is a high correlation between PSF values obtained from datasets under abovementioned condition variations. PSF analysis minimizes the systematic bias introduced by variation in experimental procedures, since PSF considers relative fold change values of gene expression in disease versus control.

### Pathway signal flow-self organizing maps analysis (PSF-SOM)

**Fig 1:**
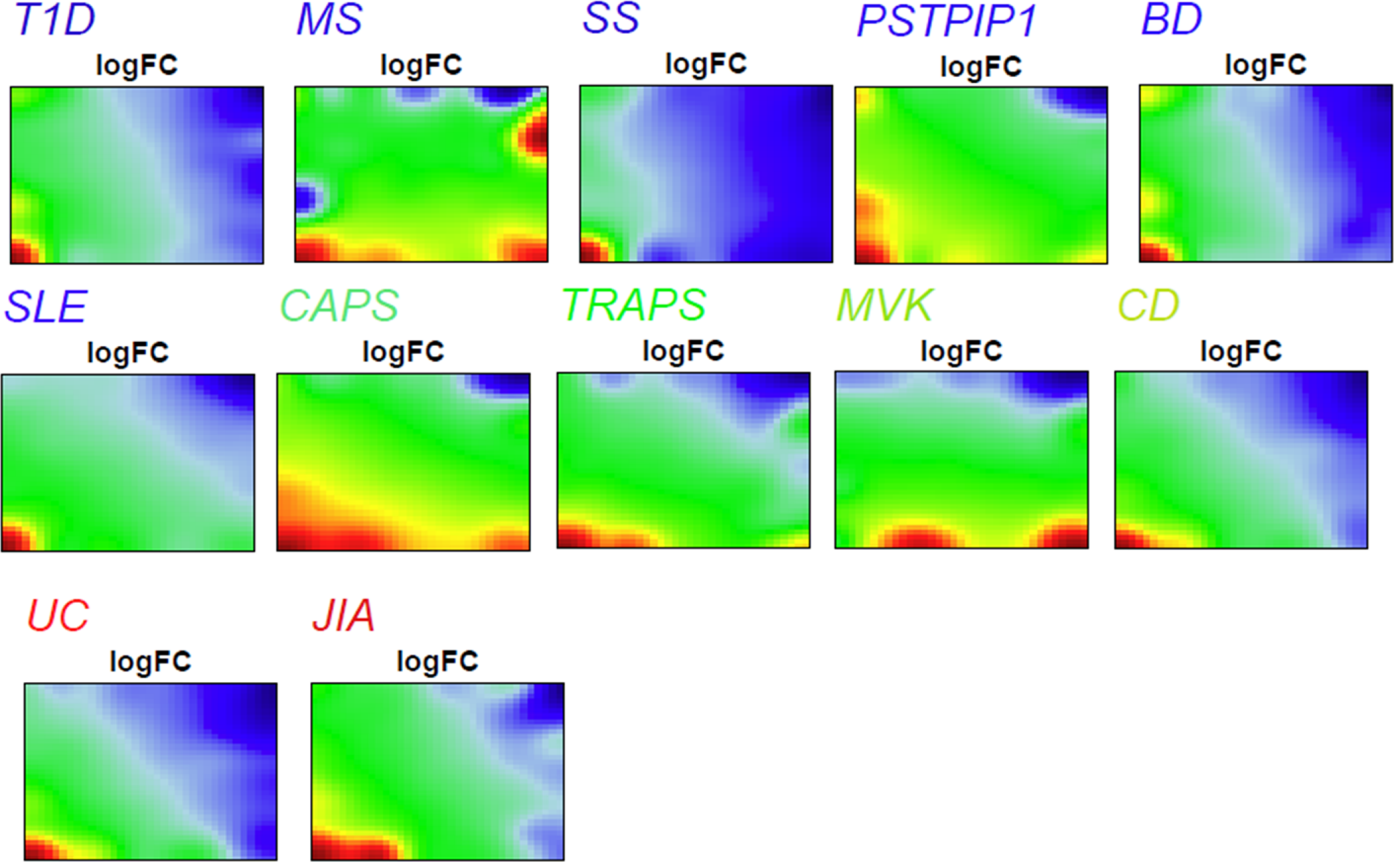
Disease specific SOM portraits. PSF profiles for each disease were mapped on a 35x35 SOM grid, and visualized as 2D maps, where colors indicate the actual state of pathway sink dysregulations. Red to green color gradient indicates up-regulation, while blue to green one indicates down-regulation.

The PSF profiles described above were clustered and visualized with the Self Organizing Maps (SOM) algorithm, implemented in the R package “oposSOM” [42,43]. This algorithm was initially intended for application to gene expression data, but has also been applied for analysis of PSF values (PSF-SOM) [38]. The PSF-SOM algorithm takes as input the sample-wise PSF profiles of the 1825 sink nodes, and aggregates the similar profiles into meta-profiles, or meta-sinks. It arranges the meta-sinks on a two-dimensional grid in a way that correlated meta-sinks are located close to each other. In our analysis, we have obtained a 35x35 grid of meta-sinks. Using the meta-sink locations on the grid, we can then obtain a SOM portrait for each sample, where the mean PSF values of the meta-sinks are visualized with blue-green-to-red color scale (Fig 1). In other words, all the SOM portraits have the same arrangement of meta-sinks, but the PSF values differ based on the sample. Clusters of co-regulated meta-sinks are then detected as so-called spots in the SOM portraits using a detection threshold described in [42].

### Spot based disease similarity assessment

The similarity between diseases was assessed based on the number of shared spots between disease pairs. For this, we have constructed a graph object, with nodes representing the individual SOM-portraits of each disease, and edges connecting the disease pairs that share the highest number of spots: in case of ties, more than one edge was introduced. Community search within this graph was performed with the walktrap algorithm implemented in the R “igraph” package [44].

### Functional annotation of sinks

Functional annotation of sink dysregulation was assessed using overrepresentation approach implemented in the WebGestalt web-tool [45,46]. The pathway sink genes were tested against Gene ontology (GO) database. The minimum number of genes in a category was set to 5, and the significance threshold to p<0.05 after Benjamini & Hochberg multiple test correction. In order to achieve higher level of outlook on the biological processes conveyed by GO terms we performed semantic-similarity based summarization and removal of redundant terms using the REVIGO tool [47].

## Results and discussion

### PSF-SOM disease portraits

The SOM method transforms the multidimensional PSF data into a series of two-dimensional images, called "portraits", which visualize the activities of the pathway sink nodes in each studied disease (Fig 1). These PSF-activity portraits show blue and red spot like regions corresponding to down- and up-regulated sink nodes, respectively. It should be noted that the portraits depict changes in pathway activities compared to the healthy state.

**Fig 2:**
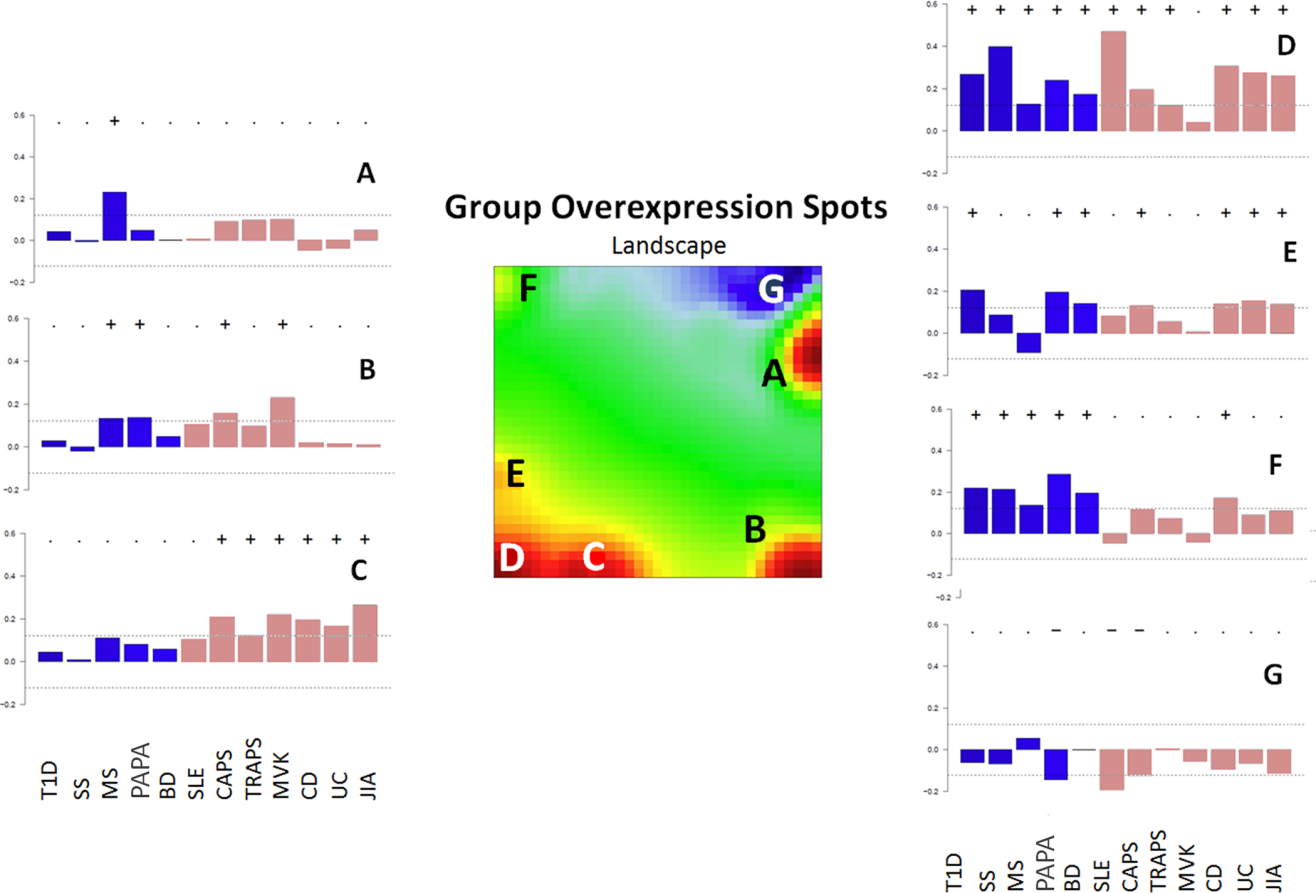
Global landscape of pathway dysregulations in diseases. The summary SOM portrait with spots transferred from individual disease portraits. The seven spots detected in the SOM portraits are assigned the letters A-G (in the center of figure). PSF-activity profile barplots (left and right panels) represent the mean PSF-signal for a given spot across the diseases. If that value passes the defined threshold the spot is marked with a “+” sign for up-regulation and “-” sign for downregulation. Bar coloring indicates the relation of diseases to similarity clusters (see Fig 3).

At-a-glance examination of the spot distribution across disease portraits revealed considerable similarities, though some differences were observed as well: almost all the diseases were characterized by the presence of upregulated spot areas at the lower left corner, except for MVK. In addition, MS had a characteristic red spot in upper right corner, while MVK, CAPS, MS and to lesser extent PAPA (PSTPIP1) were characterized by the presence of an additional spot near the bottom right corner of corresponding SOM portraits. Finally, it should be noted that three monogenic autoinflammatory diseases (CAPS, TRAPS, MVK), as well as JIA and MS, and to lesser extent UC and CD, contained an additional upregulated spot near the lower left corner of their SOM portraits. In order to proceed with comparative analysis and functional annotation, all the up- and down-regulated spots from the averaged portraits of each disease were transferred into a single summary map for an overview (Fig 2). We have identified seven spot clusters assigned with capital letters A-G, each showing a specific profile of PSF-values in the studied diseases (Fig 2). The full report of PSF-SOM is available at [48].

It should be noted that oposSOM scales data individually, before drawing the expression portraits, but applies spot detection thresholds on the global summary map, using all of the datasets at once (Fig 2). Hence, there may be inconsistency between visual perception of spot presence on the individual SOM portraits (Fig 1), and their threshold-based detection results (Fig 2) [42,43].

### Disease similarity assessment

**Fig 3:**
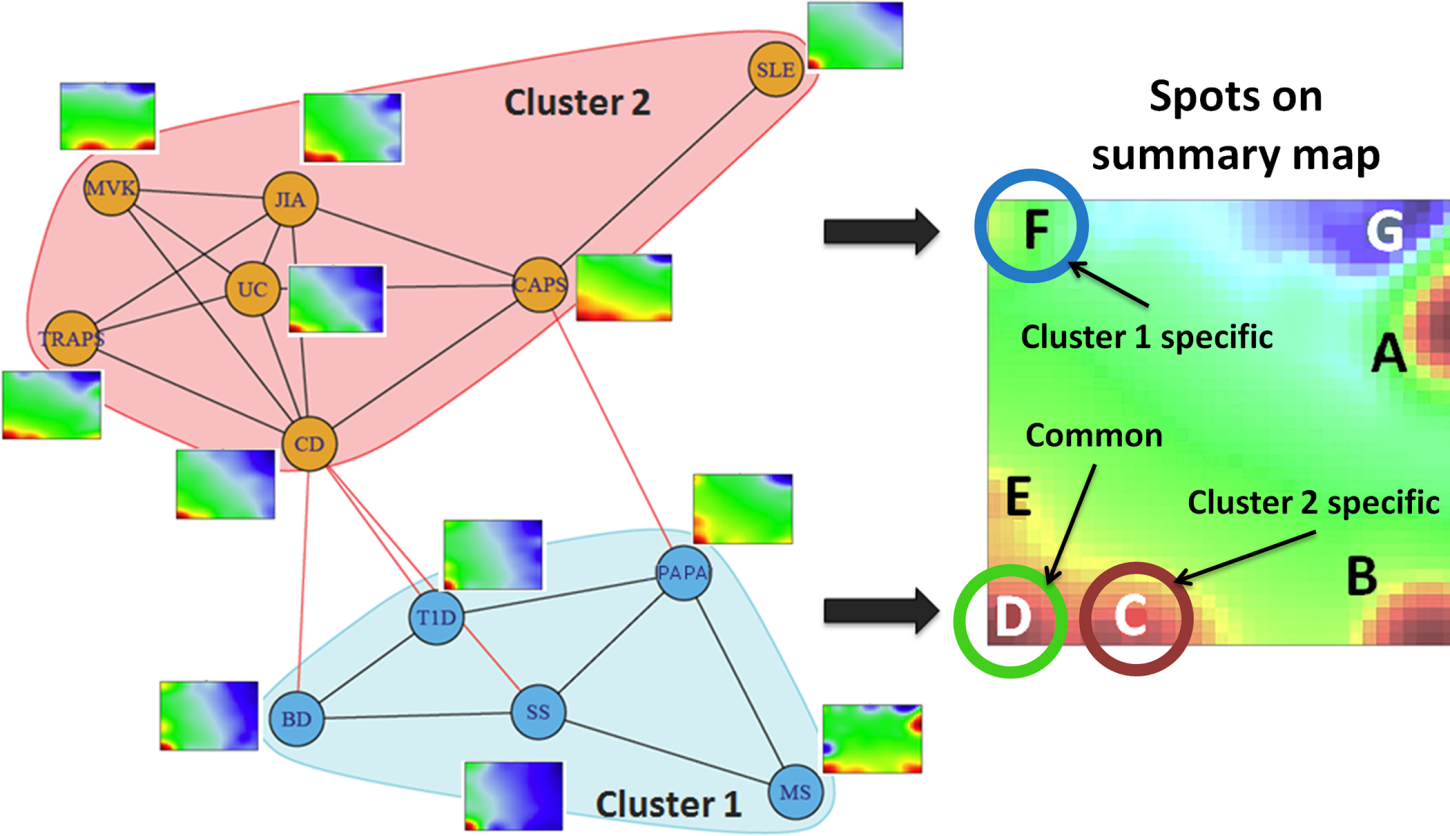
The spot-similarity graph of the diseases. The nodes represent the diseases; the edges connect the disease portraits sharing the highest number of spots. The portraits are provided as thumbnails to each node. The graph is divided into the pink and the blue clusters using the walktrap community search algorithm, implemented in R package “igraph” [44].

To assess similarities between the PSF portraits we have created a graph object where the nodes represent the diseases, and the edges connect the disease portraits sharing the highest number of spots. Based on a walktrap community detection search on this graph structure, we have identified two clusters or communities of highly similar diseases (Fig 3).

Cluster 1 contains four polygenic autoimmune diseases studied *i*.*e*. T1D, BD, SS and MS, as well as one autoinflammatory syndrome, the PAPA (PSTPIP1). This cluster is characterized by up-regulated spots D and F and collects mostly autoimmune diseases, which have been shown to largely share symptoms and clinical manifestations. Several published case reports documented that primary SS manifestations can mimic those of MS [49–51]. Moreover, SS frequently accompanies diabetes in humans [52] and in the T1D animal model of Non-Obese Diabetic (NOD) mouse [53]. Studies in the NOD mice have identified that T1D resistance genes in specific chromosomal loci (*Idd*3 and *Idd*5) protected the organism from inflammation and also of dysfunction of the salivary glands [53]. This indicates that possibly a general mechanism exists for maintaining a self-tolerant anti-inflammatory state and it affects more than one AI disease [54]. MS shares similarities with neurological manifestations of BD [55]. BD in turn has been reported to accompany T1D [56,57].

Notably, despite the fact that PAPA is an autoinflammatory syndrome and mutations in PSTPIP1 are thought to be linked to inflammasome activation and IL-1β oveproduction, PAPA lacks spot C and clusters with autoimmune diseases. The observed difference between PAPA and other autoinflammatory disorders has also been reported in the literature. So, patients with PAPA syndrome are less responsive to anti-inflammatory treatment targeted against IL-1β and TNF alpha signaling [58]. Moreover, it has been shown that in a mouse model of PAPA, PSTPIP1 did not regulate inflammasome activation, suggesting alternative effects of PSTPIP1 mutations in PAPA [59]. Furthermore, spot C collects sinks related to “actin filament-based movement”, while mutations in PSTPIP1 lead to impaired podosome (actin-containing adhesive and invasive structure) formation [60]. All in all, this result indicates that PAPA, being an autoinflammatory syndrome, has specificities in molecular events that distinguish it from other autoinflammatory disorders.

Based on unexpected clustering of PAPA with autoimmune disorders, and the observed difference between autoinflammatory diseases described by inflammasome activation, we have looked into PSF signals of NOD-like receptor signaling pathway in all the studied conditions, since it collects inflammasome related signaling events. The results show expected clustering of MVK, PAPA, TRAPS and CAPS that were separate from other diseases (S2 File). This in turn indicates that the specificities observed for PAPA syndrome are not related to inflammasome formation, but rather to dysregulation of other pathways.

Cluster 2 collects CD, CAPS, UC, JIA, TRAPS and MVK and is specifically characterized by up-regulated spots C and D (Fig 3). This cluster is more homogeneous, and consists of monogenic (MVK, CAPS, TRAPS) and polygenic (JIA, CD, UC) autoinflammatory disorders [32,61].

Surprisingly, SLE, being an autoimmune disorder, also fell into this cluster. The systemic inflammatory nature of SLE whereas the immune system attacks healthy tissues in several parts of the body may explain the shared spots with the other autoinflammatory syndromes in the cluster [62]. Recent findings indicate that SLE shares molecular signatures with type I interferon-mediated monogenic autoinflammatory disorders [63]. Moreover, there is an indication on the emerging role of the inflammasome in SLE pathogenesis [64]. Recent studies by Shin *et al* [65] and Zhang *et al* [66] demonstrated that anti-self dsDNA antibodies in monocytes can induce the activation of NLRP3 inflammasome, leading to production of IL-1β. The latter in turn induces Th17 cell response in SLE. Another study by Kahlenberg *et al* [67] has found that caspase 1, the central enzyme involved in inflammasome activation, is essential for development of SLE in a mouse model of inducible lupus.

The obtained results suggest that regardless of the disease initiation event, the downstream pathway activity profiles share considerable similarities that can be attributed to chronic inflammation, which is an essential pathological event implicated in all the studied diseases. Although this is not surprising, a formal demonstration of shared inflammation-related pathways between these diseases suggests that the observed pathway dysregulations may create conditions for the development of comorbid syndromes.

### Functional context of dysregulated sinks and pathways

**Fig 4:**
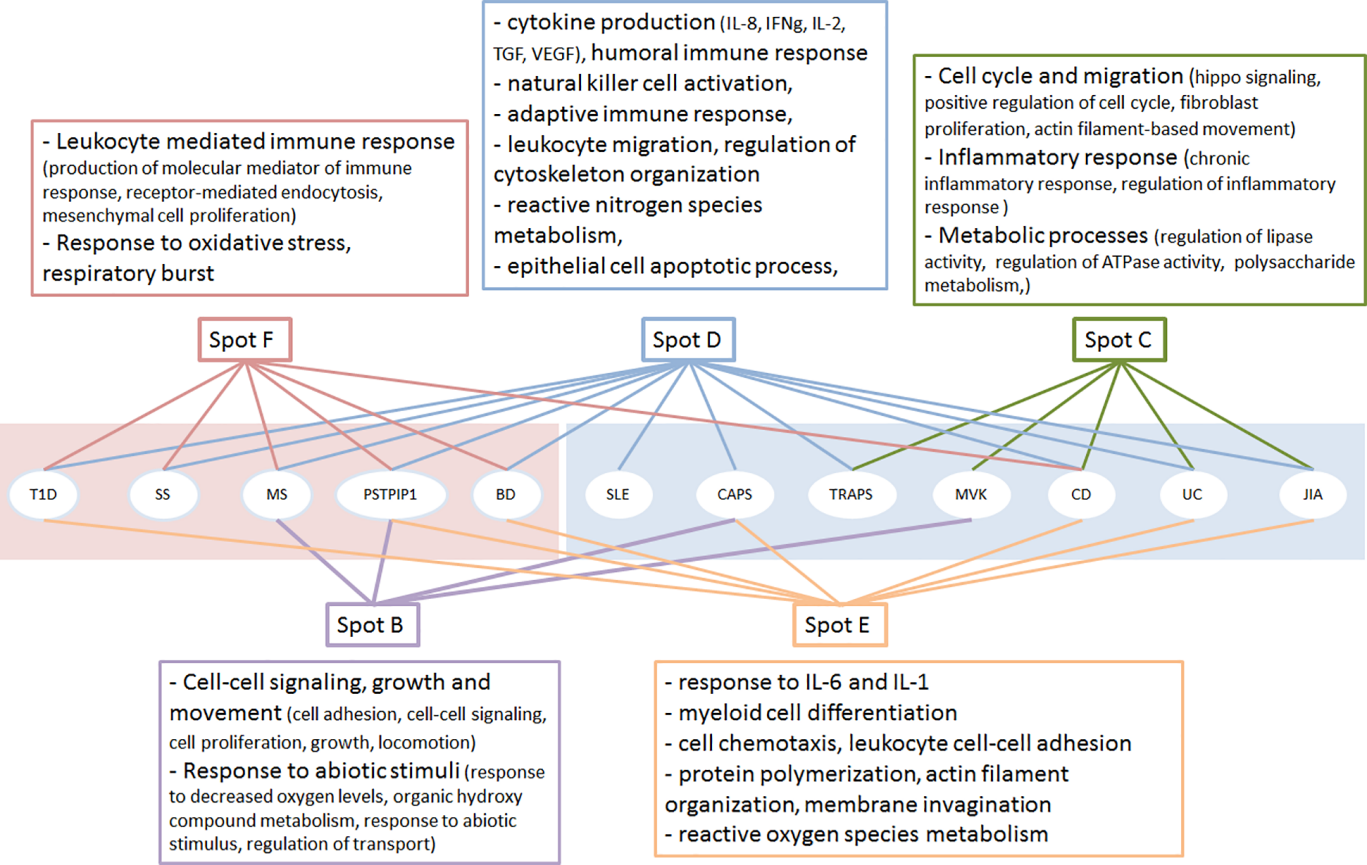
Functional annotation of biological processes associated with spots and their contribution to diseases. The pink (left) and blue (right) background indicate the two clusters of disease obtained by spot-similarity analysis. The sinks in each spot were annotated with Webgestalt and REVIGO programs to reveal associated GO term and summarize them with non-redundant descriptors.

For functional annotation of pathway sinks associated with the spots of the summary map, we have performed GO term enrichment analysis using the WebGestalt and REVIGO programs [46,47]. According to the results, all spots were enriched with GO terms related to various aspects of immune response (Fig 4). The obtained results confirm the redundancy of main pathological events leading to development of chronic inflammation, which is indicated by the presence of spot D in all the diseases (except for MVK). Meanwhile, we have also observed some degree of specificity driven by the presence of specific spots, each accumulating distinct sets of processes that were unique for each disease cluster. Spot F relevant to the disease cluster 1 (autoimmunity) shows enrichment of GO terms associated with immune response, while spot C, which is unique to the disease cluster 2 (autoinflammation) is enriched with terms associated with inflammation.

Two other spots, B and E, seem to serve as supporting nodes that are either amplifiers (as, for example, in the case of CAPS or MS) or provide alternative mechanisms for immune response activation (as in the case of MVK). The complete list of GO terms associated with the pathway sinks and the enrichment p values can be found in the S3 File.

**Fig 5:**
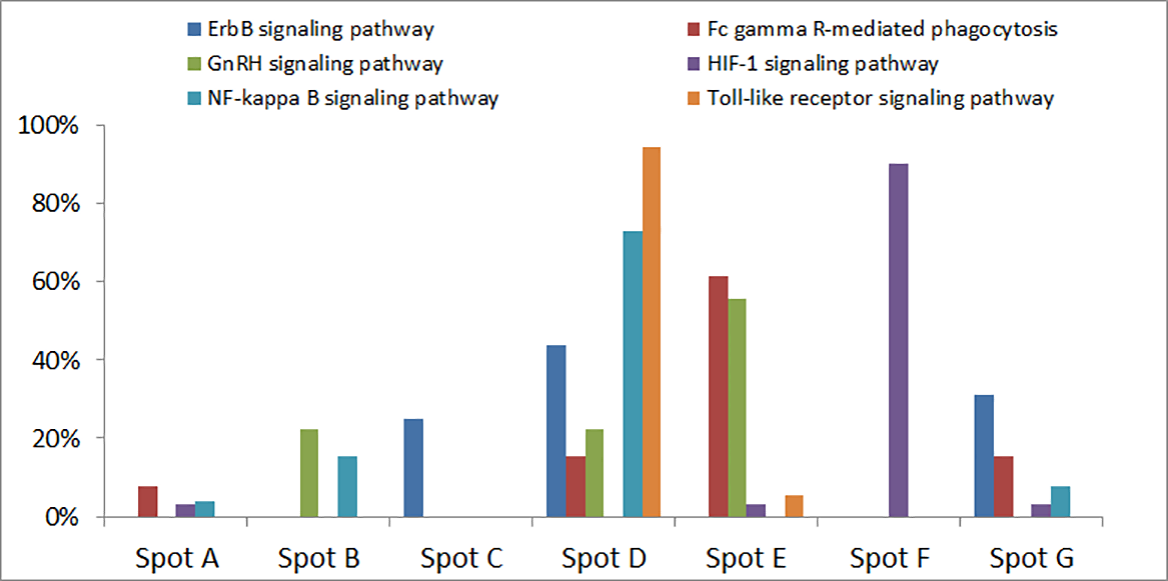
Sink distribution of top dysregulated pathways in spots. The pathways with the highest proportions of dysregulated sinks are represented in the graph. The proportion of dysregulated sinks in each spot is depicted in the heights of respective bars. Each pathway is indicated with corresponding color.

In total, 131 from 168 signaling and metabolic pathways with at least one significantly dysregulated sink in at least one disease were detected in the spots indicating that almost all pathways were dysregulated in the studied diseases (the complete list of pathway sinks is available in the S4 File). In other words, characterization of these diseases in terms of dysregulation of specific pathways seems inappropriate. Moreover, we have observed a large overlap of pathways in different spots (on average, 3 spots share sinks for a given pathway), suggesting an important role of pathway branching and the diversity of functional events associated with a single pathway (see S5 File). We have also noted that the proportion of affected branches in different pathways is markedly variable. The top pathways characterized with the highest proportion of dysregulated sinks are presented in the Table 2. Interestingly, pathway branches of top dysregulated pathways are not spread across all the spots, but are mainly concentrated in the spots C, D, E and F (Fig 5). All these pathways are directly linked to chronic inflammation, indicating their central role in the etiology of the studied diseases, as expected.

**Table 2:** Top pathways with dysregulated sinks.

Pathway	Dysregulated/Total sinks	Affected spots (% of dysregulated sinks in node)	Functional impact	References
HIF-1 signaling pathway	31/33	A(3), E(3.5), F(90), G(3.5)	links hypoxia with inflammation; switch that modulates the immune system response by regulating metabolism and apoptosis depending on the microenvironment.	[96]
NF-kappa B signaling pathway	26/28	A(4), B(15), D(73), G(8)	a major inflammatory signal transduction route; participates in immunodeficiency, autoimmunity and autoinflammation, characterized by inflammation of several organs; non-canonical branch is associated with multi-organ autoinflammation	[97–101]
Toll-like receptor signaling pathway	18/19	D(94), E(6)	part of innate immune response toward various pathogens; recognizes endogenous agents which are released upon cell damage and necrosis; a link between innate and acquired immunity and is implicated in the development of both autoimmunity and autoinflammation	([102–106]
ErbB signaling pathway	16/16	C(25), D(44), G(31)	implicated in cancer development; expression of receptors for this pathway has been reported on lymphocytes; activation leads to downstream activation of calcium, PI3K-Akt, mTOR signaling	[107–109]
Fc gamma R-mediated phagocytosis	13/14	A(8), D(15), E(62), F(15)	able to triggering both activating and inhibiting signals, leads to initiation or inhibition of a range of inflammation related events, including release of cytokines	[110]
GnRH signaling pathway	9/9	B(22), D(22), E(56)	Expression of GnRH and its receptors is detected in lymphocytes; leads to increased IL-2R; affects c-jun and ELK dependent gene expression and activation of arachidonic acid metabolism	[111,112]

In contrast to the above mentioned pathways, PI3K signaling and Axon guidance pathways had dysregulated branches in all the spots. The PI3K-Akt signaling pathway essentially serves as an integrator of signals from different pathways and controls the number of key aspects of inflammatory response, such as cell movement, growth, survival and differentiation [68,69]. Furthermore, it is known that the PI3K pathway mediates interactions between polarizing signals and glucose metabolism, as well as respiratory burst in immune cells [70].

The axon guidance pathway is implicated in formation of neuronal networks [71]. However, this pathway frequently appears in lists of pathways dysregulated in immune system related conditions [72–74]. It includes mitogen activating kinases and genes encoding proteins involved in cytoskeleton rearrangement (KEGG Pathway ID hsa04360); both essential for PBMCs to react to stimuli [75]. Moreover, it has also been demonstrated that the axon guidance genes are involved in T-cell dependent B-cell maturation [76].

Along with the expected dysregulations of immune system signal transduction pathways, our analysis points out to massive dysregulations in metabolic pathways, including energy metabolism, metabolism of biomolecules, vitamins and xenobiotics. Our results are in line with previous observations on inflammation-related metabolic changes analyzed with GWAS and metabolomics approaches [77,78]. These data demonstrate that the mechanisms of the studied diseases expand far beyond the immune system dysregulations and affect pathways that regulate the basic cell functions. It should be noted, that in contrast to immune system related pathways, dysregulations in metabolic pathways were concentrated in specific spots: 28 out of 43 metabolic pathway dysregulations were spot specific (see S5 File), compared to 4 out of 14 immune system pathways.

In order to evaluate the relevance of the observed pathway deregulations in disease context, we have also evaluated the impact of treatment and disease outcome on pathway activation profiles in the studied diseases (S6 File). Strong association was observed between the known disease-drug gene sets and the spot-related pathway sink nodes identified in our analyses. The results suggest that the output nodes associated with identified pathway perturbations in this study may eventually serve as drug targets. Finally, we have also shown that the pathways commonly perturbed in PBMCs and in target tissues of organ-specific autoimmune disorders are partly resolved after disease treatment or during disease remission stages.

### Dysregulated pathway-infrastructure in autoimmune and autoinflammatory diseases

Considering that in both AI and AIF diseases the common characteristics lie in self-directed inflammation and disturbed homeostasis of canonical cytokine cascades, a comparative analysis of the pathways involved could lead to transition to a systems level understanding of the observed phenomena. The results of previous studies based on analysis of genetic variations using genome-wide scans, gene expression analysis data and response to similar drugs, have already suggested that chronic inflammatory diseases share common mechanisms or pathways [32,79,80]. However, such studies are usually limited in the examined diseases, as well as in their attempt to obtain a general image of the mechanisms involved.

Here we have employed a systematic approach that demonstrates the universality of pathway dysregulations in autoimmune and autoinflammatory disorders, as well as uncovers novel aspects of similarity in the pathomechanisms of these diseases that were not reported previously. The results of this study clearly suggest that there is no single mechanism governing the development of the studied conditions. On one hand, the picture is rather complex and conditioned by a big number of variables. On the other hand, our pathway activity based disease clustering showed that diseases characterized by highly divergent clinical manifestations largely share the underlying pathomechanisms, at least at pathway activation levels. In some cases, disease linkage in the obtained clusters was supported by previous observations, and in others, the obtained results were novel. For example, we have addressed in our study the specifics of pathway dysregulation in PAPA [81] and HIDS (MVK) syndromes [82]. Previous studies have clearly implicated NLRP3 and pyrin inflammasomes in activating caspase-1 and IL-1β production in both syndromes [83–85]. Interestingly, while HIDS, in contrast to other autoinflammatory diseases, is caused by mutations in a metabolizing enzyme (MVK) [21,85,86], it was later demonstrated that mutations in the *MVK* gene disrupt the action of mevalonate kinase (MVK) resulting in a cascade of processes that involve pyrin inflammasome activation and enhanced IL-1β secretion [83,87]. This may partially explain the difference in spot enrichment observed in this disease. However, less is known about dysregulation states of other pathways involved in pathogenesis of these disorders. Our data suggest that PAPA is characterized by pathway activation patterns close to that of autoimmune diseases (Fig 3, cluster 1), while HIDS clusters with the classical autoinflammatory syndromes (Fig 3, cluster 2). Our analysis allowed us to move beyond the traditional gene-centered approach of description of molecular pathomechanisms, and to summarize the diseases at a systems biology level. Compared to the popular Gene Set Enrichment Analysis, PSF-SOM was able to mine far more functional information [38], providing a global picture of self-directed inflammatory syndromes and generate novel hypothesis for data validation to be undertaken in additional studies.

### Advantages and limitations of PSF-SOM analysis of PBMC

Our aim in this study was to compare diseases that share self-inflammatory phenotypes without prior restriction for taking in consideration genetic determinants specific to each disease. Therefore the most appropriate approach was the comparative analysis of existing microarray transcriptome data by our PSF-SOM method, allowing to tackle quantitative variations in pathways rather than in single genes. We appreciate that utilization of RNA sequencing data would further improve the analysis results, as it has a number of advantages over microarrays and even more valuable results could be obtained with the use of single cell transcriptome data. Unfortunately, current availability of RNA sequencing data for cell subpopulations is limited, and it is impossible to collect homogeneous data in terms of sequencing platform and sample types for all the studied diseases. Single cell data are even scarcer and still possess technological and algorithmic challenges [88]. However, our approach is technology independent and makes it possible to replicate the study once such data is available.

In our analysis we have considered only PBMC. While this choice offers data homogeneity, together with the facility of a non-invasive and easily obtainable tissue, it may reveal inflammatory-related and/or other unrelated processes, reflecting a steady state of the corresponding organism including influences from a disease-unrelated environmental status. However, taking into consideration the concept of an immunological continuum, whereas tissue perturbations at the target sites of inflammation, rather than the immune system *per se*, are the key to disease expression, such perturbations may be reflected in the peripheral blood. Previous reports of transcriptome analysis in the peripheral blood have demonstrated that it is a well suited surrogate tissue, reflecting distal organismal perturbations and providing a large sensitive pool of gene transcripts with quantitative fluctuations detectable as gene expression modifications [89].

In order to show if the processes identified in PBMC at least to some extent reflect the processes activated in the target tissues, we have performed additional analysis to explore the overlap of pathway activities between PBMCs and target tissues for organ-specific autoimmune disorders, namely, diabetes type 1, multiple sclerosis, Crohn’s disease and ulcerative colitis, where immune responses are directed against antigens present in a particular organ (S7 File). We have found that the activated pathways in PBMCs and in target tissues considerably overlap (34–59%, depending on the disease). This suggests that the processes identified in PBMC show common features with the disease characteristics of the tissue lesions.

It is worth pointing out that the results of this study should be interpreted with some degree of caution. The difference in the number of samples for each condition may lead to uneven variation of pathway activity values across datasets, which may bias the interpretation of the results. Another issue concerns the fact that according to the dataset descriptions available in the GEO, the samples were collected at different stages of disease progression, and not at the stage of initiation, thus making it perhaps more difficult to identify disease-specific determinants. Indeed, the initiating factors of each disease can be easier identified at the early stages, while those can be masked at later stages of the disease. This could lead to clustering of diseases otherwise being of different molecular origin. Nevertheless, the difficulty of collecting human tissues at early disease time points, due to the absence of early phenotypic landmarks and/or known early markers, renders such studies not feasible for the moment. A few studies have addressed this issue in both AI and AIF disorders [90–92]. One promising approach for the identification of non-invasive biomarkers is the exploration of microRNAs easily accessible in many body fluids [93].

## Summary and conclusions

The data presented in this report support the notion that diseases could be distinguished by pathways of the adaptive or innate immune responses, with the majority of conditions connecting by variable degrees of interaction between these two systems [94,95]. Our previous study on lung diseases using a similar systems biology approach has resulted in clear separation between cancer and other chronic lung diseases, each group characterized by a specific set of dysregulated pathways [38]. The separation we observed in that study was attributed to a distinct set of pathomechanisms implicated in cancers (cell proliferation, metabolism) and other lung diseases (immune/inflammatory response and fibrotic tissue remodelling) [38]. Here we have limited our scope to study only immune-system related diseases, and expected to get higher degree of interrelatedness at the level of pathway activity perturbations, compared to the previous study.

Overall, our study offers a systems biology approach addressing the complexity and redundancy of the immune system mechanisms implicated in the inflammatory origin of the pathogenesis of the studied diseases. Identification of pathways shared between different diseases, has the potential to generate novel hypotheses and elaborate methods to subvert the immune dysfunction by modulating networks through regulation of one gene or miRNAs. Moreover, it may contribute to our understanding of similarities and differences in responses to similar treatment depending on specific patterns of pathway alterations. The selection of pathway level resolution provides a more aggregated level of information, as compared to single gene level analysis. We believe that both gene- and pathway- level analyses are important, one complementing the other.

## Supporting information

S1 File Analysis for batch effects and platform independence in PSF calculations. (DOCX)

S2 File Analysis of inflammasome activation. (DOCX)

S3 File GO term enrichment. (XLSX)

S4 File Sink-spot association. (XLSX)

S5 File Distribution of pathway sinks across spots. (XLSX)

S6 File Treatment effect on pathway activation profiles. (DOCX)

S7 File Analysis of pathway activation profile overlaps between tissue specific and systemic response. (DOCX)
