# Supplementary material for: Autoimmunity and autoinflammation: A systems view on signaling pathway dysregulation profiles
Source: PLoS One. 2017 Nov 3;12(11):e0187572. doi: 10.1371/journal.pone.0187572 (PMC5669448; doi:10.1371/journal.pone.0187572)
Supplement: S1 File — (DOCX) [file pone.0187572.s001.docx]

# S1 Supporting information

# Analysis of batch effects and platform independence in PSF calculations

In order to test whether the PSF calculation approach may be beneficial in overcoming batch effects we performed analysis using datasets obtained by different platforms or different experiments for the same disorders.

Maouche et al [1] published the paper on concordance of microarray gene expression measurement in Affymetrix and Illumina platforms. In their experiments they assessed the gene expression in macrophages and resting monocytes 5 from patients with acute coronary syndrome. RNA samples for each type of cell were hybridized to the two platforms in parallel. Datasets are accessible through GEO Series accession number GSE10213 (Illumina data) GSE11430 (Affymetrix data) and containing log-transformed and normalized gene expression values. In their paper, the authors have shown that while raw gene expression values do not correlated between batches, the fold change values do. We proceeded with correlation analysis on PSF values.

PSF values were calculated as described in S7 Supporting information using gene-wise mean expression of monocytes as reference. Our analysis indicated strong correlation between PSF values calculated with gene expression data from different platforms (Figure S1.1).


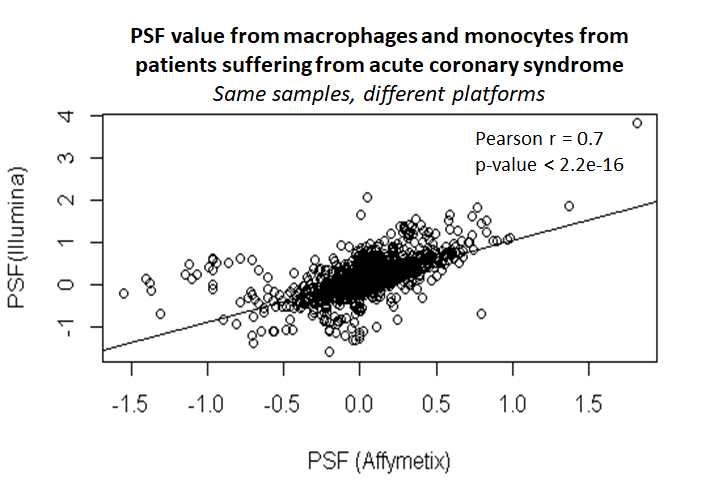


**Figure S1.1.** Correlation between PSF values for gene expression datasets obtained from the same samples (macrophages and resting monocytes from 5 patients), but with different microarray platforms. The Pearson correlation coefficient of PSF values was 0.7, p-value < 2.2e-16.

In addition, we have evaluated the concordance of PSF values computed from gene expression data from different platforms obtained from PBMCs of patients suffering from Sjogren's syndrome. We used the dataset GSE48378 included in our original analyses and the dataset GSE84844 publicly released in May 19, 2017 (Figure S1.2).


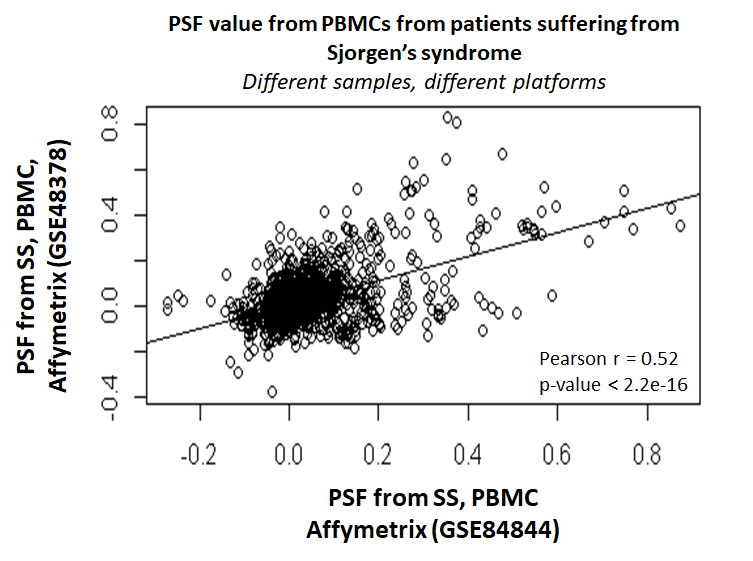


**Figure S1.2.** Correlation between PSF values for gene expression datasets obtained from PBMCs of patients suffering from Sjorgen’s syndrome fromdifferent samples and with different microarray platforms. The Pearson correlation coefficient of PSF values was 0.52, p-value < 2.2e-16.

Finally, we have also evaluated the effect of experimental batches on gene expression values, by comparing the PSF values for gene expression datasets obtained by the same platform, but by different research groups. For this we have used the dataset GSE67596,

included in our original analyses and the dataset GSE21521, both containing gene expression profiles in juvenile idiopathic arthritis measured with Affymetrix Human Genome U133 Plus 2.0 Array (Figure S1.3).


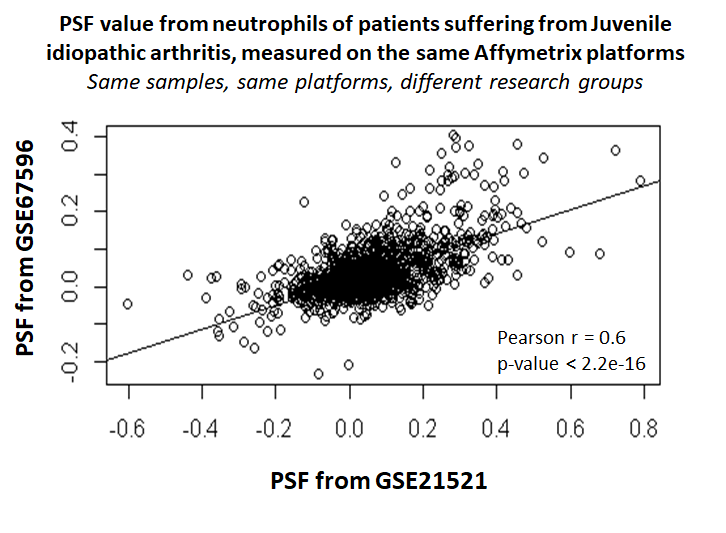


**Figure S1.3.** Correlation between PSF values for gene expression datasets obtained from neutrophils of patients suffering from juvenile idiopathic arthritis from the same samples, the same microarray platforms, but with different research groups. The Pearson correlation coefficient of PSF values was 0.6, p-value < 2.2e-16.

Overall, these three analyses demonstrate that PSF values have considerable correlation despite difference in experiments, platforms and samples. This means that the possible batch effects that could arise from the mentioned differences are minimized by PSF calculations based on gene expression fold changes, and that using PSF values from different experiments in a meta-analysis is a valid approach.

**Refrences**

1. Maouche S, Poirier O, Godefroy T, Olaso R et al. Performance comparison of two microarray platforms to assess differential gene expression in human monocyte and macrophage cells. BMC Genomics 2008 Jun 25;9:302. PMID: 18578872
