# Supplementary material for: Autoimmunity and autoinflammation: A systems view on signaling pathway dysregulation profiles
Source: PLoS One. 2017 Nov 3;12(11):e0187572. doi: 10.1371/journal.pone.0187572 (PMC5669448; doi:10.1371/journal.pone.0187572)
Supplement: S2 File — (DOCX) [file pone.0187572.s002.docx]

S2 Supporting information

Analysis of inflammasome activation

Autoimmunity, autoinflammation and inflammation: a systems view on signaling pathway deregulation profiles

Arsen Arakelyan*, Lilit Nersisyan, David Poghosyan, Lusine Khondkaryan, Anna Hakobyan, Henry Löffler-Wirth, Evie Melanitou and Hans Binder

* Correspondence: Arsen Arakelyan: [aarakaleyan@sci.am](mailto:aarakaleyan@sci.am)

In order to evaluate pathway activities related to inflammasome signaling in the studied datasets, we have analyzed the KEGG NOD-like receptor signaling pathway. This pathway represents the molecular processes and players involved in formation and activation of various types of inflammasomes (Figure S2 1).


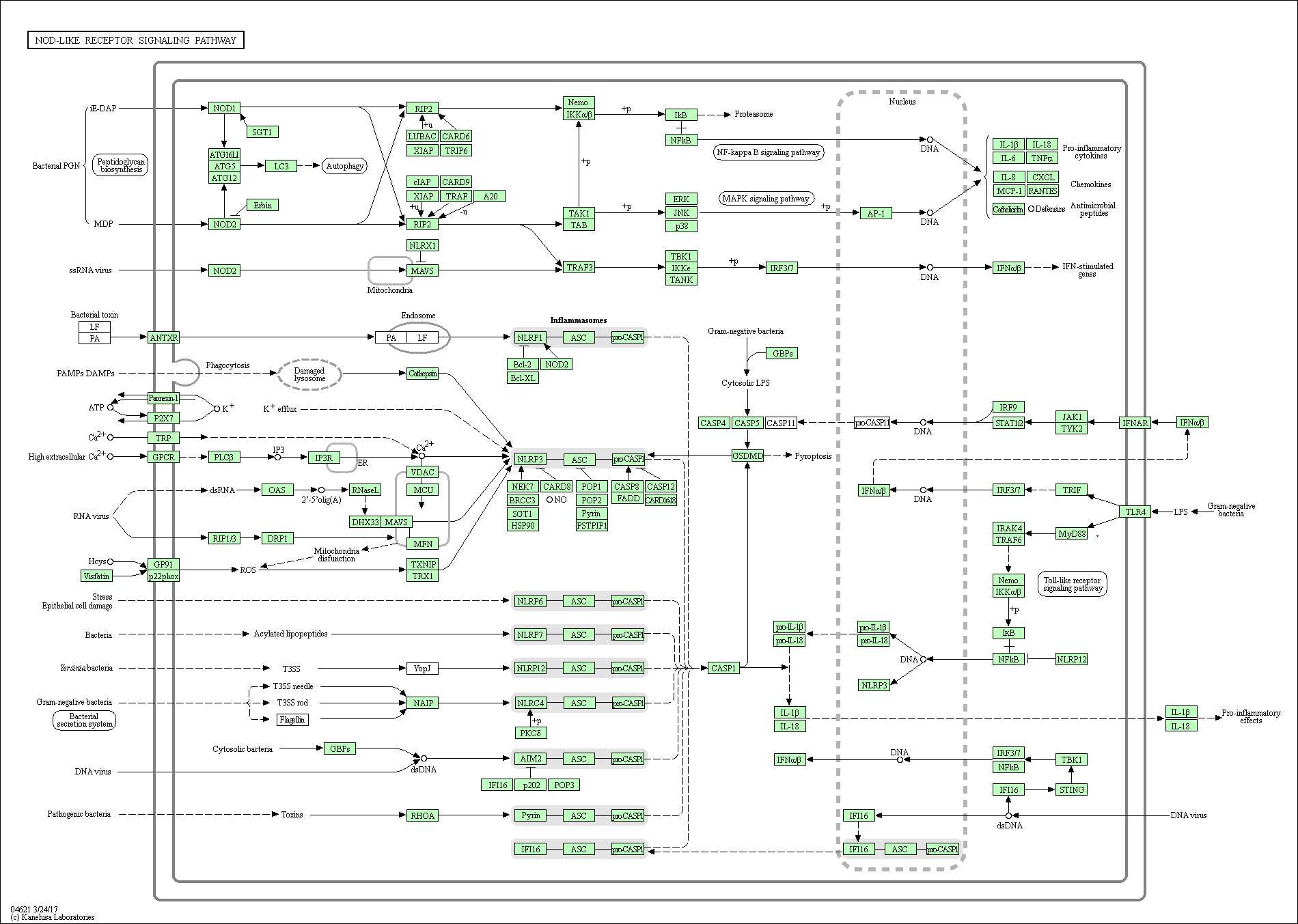


Figure S2 1. KEGG NOD-like receptor signaling pathway map. This map combines signaling from various pattern recognition receptors driving the activation of NF-kB and MAPK signaling, as well as assembly, activation of inflammasomes and inflammasome mediated IL-1β production. For high resolution image and pathway annotation refer to: <http://www.kegg.jp/kegg-bin/show_pathway?map=hsa04621&show_description=show>.

PSF values of all the nodes in this pathway in each dataset were calculated as described in the paper. The results reveal four clusters of diseases, with MVK being in separate group, and TRAPS, CAPS and PSTPIP1 combining in one cluster. JIA, DM, CD and UC form the third cluster, while MS, SS, BD and SLE for the last one. According to the heatmap, the last two clusters have down regulated PSF values in the nodes involved in inflammasome formation, while MVK, and TRAP, CAPS, PSTPIP1 have upregulated values in the upper part of the heatmap.


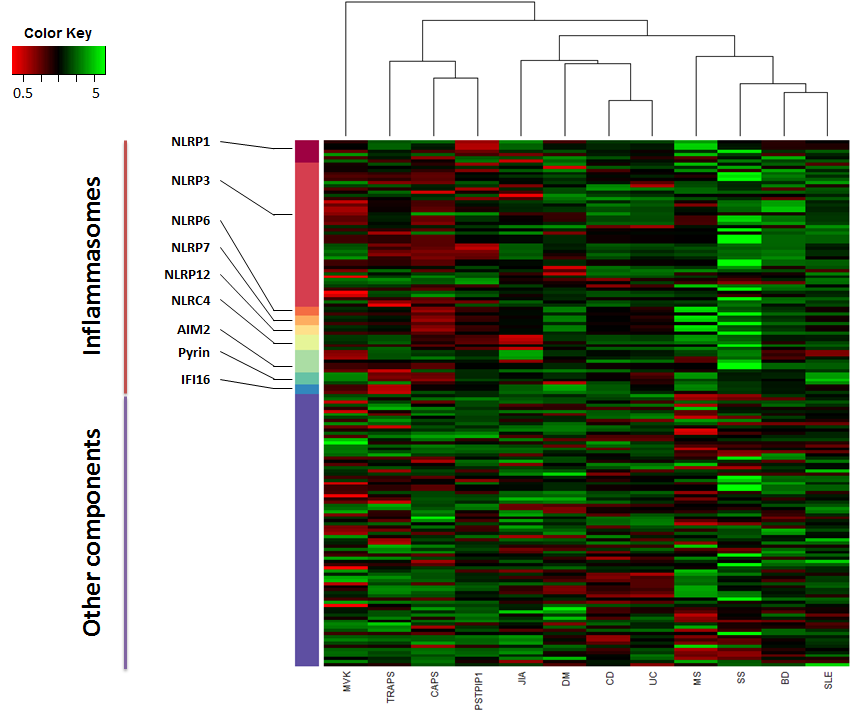


Figure S2 2. Heatmap of PSF values of all the nodes in the NOD-like receptor signaling pathway. The nodes are order according to their direct involvement in the formation of various types of inflammasomes (upper part, various colors), and the rest of the nodes in the pathway are placed in the bottom part of the heatmap (violet). The datasets are labeled in the bottom of the heatmap.

The PAPA syndrome (labeled by PSTPIP1) clusters with autoinflammatory disorders according to inflammasome activation. Therefore, its clustering with autoimmune diseases that was reported in the main paper is due to involvement of other pathways. Additionally, while MVK, and TRAP, CAPS, PSTPIP1 show activation of inflammasomes, it can be noticed that they differ in the type of inflammasome activated.
