# Supplementary material for: Autoimmunity and autoinflammation: A systems view on signaling pathway dysregulation profiles
Source: PLoS One. 2017 Nov 3;12(11):e0187572. doi: 10.1371/journal.pone.0187572 (PMC5669448; doi:10.1371/journal.pone.0187572)
Supplement: S6 File — (DOCX) [file pone.0187572.s006.docx]

# S6 Supporting information Treatment effect on pathway activation profiles

We performed additional analyses to evaluate the impact of treatment and disease outcome on pathway activation profiles in studied diseases. Three datasets (table S6.1) were downloaded from GEO and preprocessed as it was described in S5 Supporting information. The PBMC datasets for these diseases were analyzed as described in the manuscript. We have compared the up-regulated pathways profile overlaps between treated and non-treated samples with PBMCs.

**Table S6.1** Description of tissue specific datasets

| GEO Accession | Disease | Tissue | Samples (number-description) |
| --- | --- | --- | --- |
| GSE17635 | Type 1 diabetes | endothelial progenitor cell | 11 - non-treated  10 - treated with folic acid  11 - healthy controls |
| GSE41890 | Multiple sclerosis | whole blood | 22 - disease relapse  22 - disease remission  24 - healthy subjects |
| GSE52746 | Crohn’s Disease | colon biopsy | 10 - active CD  5 - active CD treated with anti-TNF-a  7 - inactive CD treated with anti-TNF-a  17 - healthy controls |

The results are presented in Venn diagrams and tables below. In all the cases, the overlap between PBMC and the target tissue is bigger than in the target tissue in the non-treated case, compared to the treated samples or those in remission. In other words, some of the up-regulated pathways that are common between PBMCs and target tissues get resolved after treatment or due to disease remission. This supports the hypothesis that pathways identified in PBMC partially reflect the processes occurring in the target tissue.

## Type I diabetes

| **Not treated** | **Treatment with folic acid** |
| --- | --- |
| 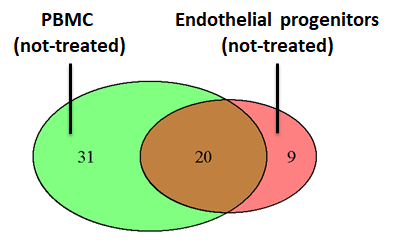  **Figure S6.1.** The venn diagram showing the overlap between up-regulated pathways in PBMCs (green) and in endothelial progenitors (red) in type I diabetes. | 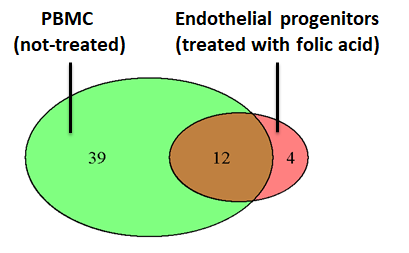  **Figure S6.2.** The venn diagram showing the overlap between up-regulated pathways in PBMCs (green) and in endothelial progenitors (red) in type I diabetes after treatment with folic acid. |
| **Table S6.1.** Commonly up-regulated pathways in PBMCs and endothelial progenitors in type I diabetes before and after treatment. Pathways resolved after treatment are highlighted in yellow on the left, new pathways appearing after treatment are highlighted in yellow on the right. | |
| 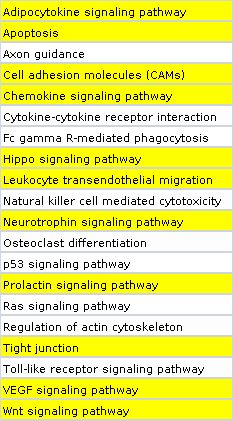 | 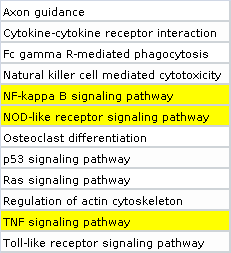 |

## Multiple sclerosis

| **Disease relapse** | **Disease remission** |
| --- | --- |
| 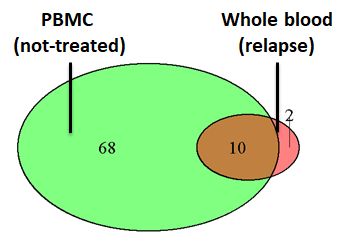  **Figure S6.3.** The venn diagram showing the overlap between up-regulated pathways in PBMCs (green) and in whole blood of samples in the relapse of multiple sclerosis. | 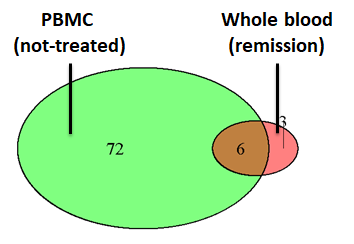  **Figure S6.4.** The venn diagram showing the overlap between up-regulated pathways in PBMCs (green) and in whole blood of samples in remission stage of multiple sclerosis. |
| **Table S6.2.** Commonly up-regulated pathways in PBMCs and whole blood of multiple sclerosis patients in the relapse and remission stages of the disease. Pathways resolved during remission are highlighted in yellow on the left, pathways activated during remission are highlighted in yellow on the right. | |
| 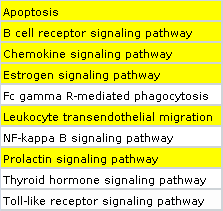 | 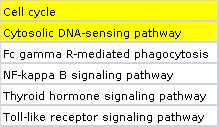 |

##

##

## Crohn’s disease

| **Active Crohn's disease** | **Active Crohn's disease treated with anti-TNF-α therapy** |
| --- | --- |
| 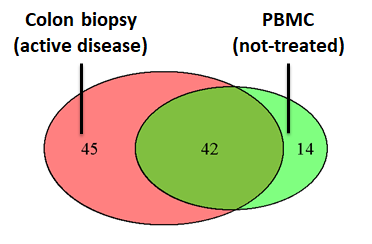  **Figure S6.5.** The venn diagram showing the overlap between up-regulated pathways in PBMCs (green) and in colon tissue of Crohn’s disease patients. | 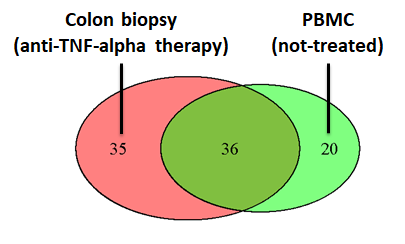  **Figure S6.6.** The venn diagram showing the overlap between up-regulated pathways in PBMCs (green) and in colon tissue of Crohn’s disease patients after anti-TNF-alpha therapy. . |
| **Table S6.3 (below).** Commonly up-regulated pathways in PBMCs and colon tissue of Crohn’s disease patients before and after treatment with anti-TNF alpha therapy. Pathways resolved after treatment are highlighted in yellow on the left, pathways activated after treatment are highlighted in yellow on the right. | |
| 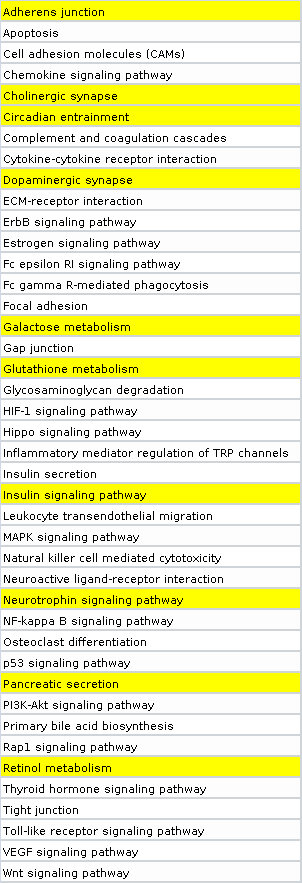 | 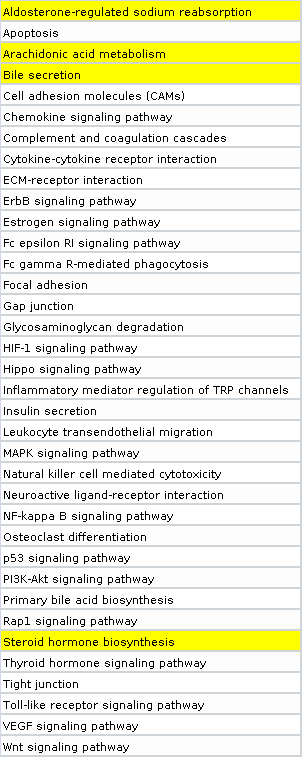 |

In addition we screened whether nodes identified in current analyses are associated with drug response gene sets for selected diseases using GeneSetDB (http://www.genesetdb.auckland.ac.nz/) and Comparative toxicogenomics (http://ctd.mdibl.org/) databases. First, we populated the list of chemicals and drugs associated with each disease from Comparative toxicogenomics database. Next, we performed overrespresentation analysis to identify significant drug associated gene sets among pathway sink nodes for each spot (FDR < 0.05 ). These lists were then compared to the disease-drug association list to find the overlaps.

Our results indicated the output nodes associated with perturbed pathways serve as a molecular targets for appropriate therapeutics (Table S6.2).

**Table S6.2** **Drug-disease-sink associations in the studied diseases.** Pathway sinks were taken from each spot, the diseases having spot enrichment were chosen, and drug association analyses were performed for the sink-set and the disease, and the overlapping hits presented in the table.

| **Spot** | **Disease** | **Drugs associated with spots and used in disease therapeutics** |
| --- | --- | --- |
| Spot C | CD | Heparin, Sulfasalazine, Thalidomide, Acetaminophen, Prednisolone |
| Spot C | JIA | Aspirin, Indomethacin, Sulfasalazine, Chloroquine, Prednisolone |
| Spot C | UC | esculetin, Nicotine, Capsaicin, Clioquinol, Sulfasalazine, Thalidomide, Prednisolone |
| Spot D | BD | Thalidomide, Sulfasalazine, Colchicine, Tacrolimus, Cyclophosphamide, Chlorambucil, Prednisone, Prednisolone |
| Spot D | CD | Thalidomide, Sulfasalazine, Heparin, Budesonide, Ciprofloxacin, Hydrocortisone, Acetaminophen, Prednisone, Prednisolone, Methotrexate |
| Spot D | JIA | Aspirin, Indomethacin, Sulfasalazine, Chloroquine, Prednisone, Naproxen, Prednisolone, Flurbiprofen, Methotrexate |
| Spot D | MS | Tacrolimus, Cladribine, Minocycline, Cyclophosphamide, Prednisone, Mitoxantrone, Prednisolone |
| Spot D | SLE | Thalidomide, Aspirin, triptolide, Indomethacin, Pentoxifylline, Tacrolimus, Diclofenac, Heparin, Ibuprofen, Glucocorticoids, Melatonin, Chloroquine, Ranitidine, Cyclophosphamide, Chlorambucil, Vincristine, Prednisone, Prednisolone, Methotrexate |
| Spot D | SS | Prednisolone |
| Spot D | T1D | Lipopolysaccharides |
| Spot D | UC | Nicotine, Thalidomide, Capsaicin, rosiglitazone, Sulfasalazine, esculetin, Budesonide, phenethyl isothiocyanate, Prednisolone |
| Spot E | BD | Sulfasalazine, Thalidomide, Colchicine |
| Spot E | CD | Sulfasalazine, Thalidomide |
| Spot E | JIA | Aspirin, Sulfasalazine, Flurbiprofen |
| Spot E | T1D | Lipopolysaccharides |
| Spot E | UC | esculetin, Nicotine, Plant Extracts, Sulfasalazine, Capsaicin, Clioquinol, Thalidomide, rosiglitazone |
| Spot F | BD | Prednisolone, Cyclophosphamide, Chlorambucil, Thalidomide, Tacrolimus, Sulfasalazine |
| Spot F | MS | Minocycline, Prednisolone, Cyclophosphamide, Tacrolimus |
| Spot F | SS | Prednisolone |
| Spot F | T1D | Lipopolysaccharides |
| Spot G | SLE | Aspirin, Tacrolimus, Melatonin, Cyclophosphamide, Diclofenac, Thalidomide, Pentoxifylline, Indomethacin, Glucocorticoids, Chloroquine, Heparin, Chlorambucil |
