# Supplementary material for: Autoimmunity and autoinflammation: A systems view on signaling pathway dysregulation profiles
Source: PLoS One. 2017 Nov 3;12(11):e0187572. doi: 10.1371/journal.pone.0187572 (PMC5669448; doi:10.1371/journal.pone.0187572)
Supplement: S7 File — (DOCX) [file pone.0187572.s007.docx]

S7 Supporting information
Analysis of pathway activation profile overlaps between tissue specific and systemic response

In order to check whether the pathways identified in PBMC were also activated in the target tissues of organ-specific autoimmune disorders, we have downloaded gene expression datasets from target tissue samples for type 1 diabetes (T1D), multiple sclerosis (MS), ulcerative colitis (UC), Sjogren's syndrome (SS), which were available in GEO (Table S7.1).

Table S7.1 Description of tissue specific datasets

| GEO Accession ID | Disease | Abbreviation | Tissue |
| --- | --- | --- | --- |
| GSE72492 | Type I diabetes | T1D | Pancreatic tissue |
| GSE26927 | Multiple sclerosis | MS | Brain tissue |
| GSE36807 | Crohn's disease | CD | Colon biopsy |
| GSE36807 | Ulcerative colitis | UC | Colon biopsy |
| GSE40568 | Sjogren's syndrome | SS | Salivary gland |

We have downloaded gene expression matrix files, performed reverse-log transformation if data were in log scale, and computed the fold change values of disease versus control samples, taking as reference the the gene-wise mean expression values of all the controls. The fold changes were then used for pathway signal flow (PSF) analysis in the same way, as performed for PBMCs. Then, the pathway-wise mean PSF values for diseased samples in PBMCs and target tissues were compared, to identify overlapping up-regulated pathways, by setting an arbitrary threshold of 1.5-fold PSF difference in disease versus controls.

The size of overlap was 42% for T1D, 59% for MS, 34% for CD, 47% for UC and 53% for SS. The Venn diagrams and the pathways found in the overlaps are presented in the figures and tables below. The results suggest that there is considerable overlap in pathway activation profiles between PBMCs and target tissues of organ-specific autoimmune disorders.

## Type I diabetes


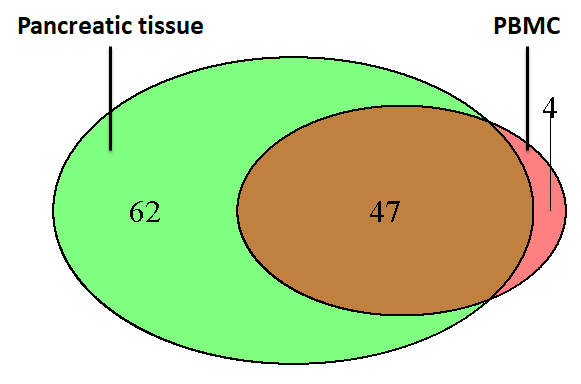


**Figure S7.1.** The venn diagram showing the overlap between up-regulated pathways in PBMCs (red) and in Pancreatic tissue (green) in type I diabetes. The up-regulated pathways have been filtered with PSF cut-off >= 1.5.

**Overlapping upregulated pathways**

| Adipocytokine signaling pathway | Intestinal immune network for IgA production |
| --- | --- |
| Adrenergic signaling in cardiomyocytes | Leukocyte transendothelial migration |
| Apoptosis | Long-term potentiation |
| Arachidonic acid metabolism | MAPK signaling pathway |
| Axon guidance | Melanogenesis |
| B cell receptor signaling pathway | Natural killer cell mediated cytotoxicity |
| Cardiac muscle contraction | Neurotrophin signaling pathway |
| Cell adhesion molecules (CAMs) | NF-kappa B signaling pathway |
| Chemokine signaling pathway | NOD-like receptor signaling pathway |
| Complement and coagulation cascades | Notch signaling pathway |
| Cytokine-cytokine receptor interaction | Osteoclast differentiation |
| Dopaminergic synapse | p53 signaling pathway |
| ErbB signaling pathway | PI3K-Akt signaling pathway |
| Estrogen signaling pathway | Prolactin signaling pathway |
| Fc epsilon RI signaling pathway | Pyrimidine metabolism |
| Fc gamma R-mediated phagocytosis | Ras signaling pathway |
| FoxO signaling pathway | Regulation of actin cytoskeleton |
| Galactose metabolism | Tight junction |
| Glycine, serine and threonine metabolism | TNF signaling pathway |
| GnRH signaling pathway | Toll-like receptor signaling pathway |
| HIF-1 signaling pathway | Tryptophan metabolism |
| Hippo signaling pathway | Vascular smooth muscle contraction |
| Insulin signaling pathway | VEGF signaling pathway |
|  | Wnt signaling pathway |

##

##

## Multiple Sclerosis


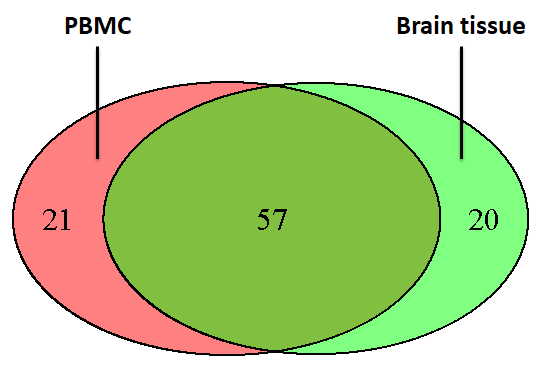


**Figure S7.2.** The venn diagram showing the overlap between up-regulated pathways in PBMCs (red) and in brain tissue (green) in multiple sclresis. The up-regulated pathways have been filtered with PSF cut-off >= 1.5.

**Overlapping upregulated pathways in MS**

| Adipocytokine signaling pathway | Insulin secretion |
| --- | --- |
| Adrenergic signaling in cardiomyocytes | Insulin signaling pathway |
| Antigen processing and presentation | Intestinal immune network for IgA production |
| Apoptosis | Leukocyte transendothelial migration |
| Arachidonic acid metabolism | Long-term potentiation |
| Axon guidance | MAPK signaling pathway |
| B cell receptor signaling pathway | Melanogenesis |
| Cell adhesion molecules (CAMs) | Metabolism of xenobiotics by cytochrome P450 |
| Cell cycle | mTOR signaling pathway |
| Chemokine signaling pathway | Neuroactive ligand-receptor interaction |
| Circadian entrainment | Neurotrophin signaling pathway |
| Complement and coagulation cascades | NF-kappa B signaling pathway |
| Cytokine-cytokine receptor interaction | Osteoclast differentiation |
| Cytosolic DNA-sensing pathway | Ovarian steroidogenesis |
| Dopaminergic synapse | p53 signaling pathway |
| ECM-receptor interaction | PI3K-Akt signaling pathway |
| ErbB signaling pathway | Progesterone-mediated oocyte maturation |
| Estrogen signaling pathway | Prolactin signaling pathway |
| Fc epsilon RI signaling pathway | Rap1 signaling pathway |
| Fc gamma R-mediated phagocytosis | Ras signaling pathway |
| Focal adhesion | Salivary secretion |
| FoxO signaling pathway | Sulfur metabolism |
| Gap junction | T cell receptor signaling pathway |
| Gastric acid secretion | Taste transduction |
| Glutamatergic synapse | Thyroid hormone signaling pathway |
| GnRH signaling pathway | Tight junction |
| HIF-1 signaling pathway | TNF signaling pathway |
| Hippo signaling pathway | Toll-like receptor signaling pathway |
|  | Wnt signaling pathway |

## Crohn's Disease


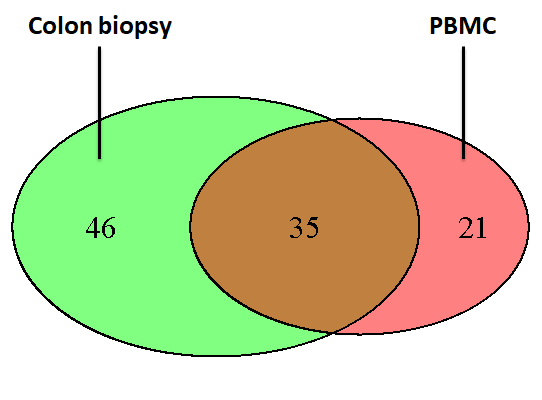


**Figure S7.3.** The venn diagram showing the overlap between up-regulated pathways in PBMCs (red) and in colon tissue (green) in Crohn's disease. The up-regulated pathways have been filtered with PSF cut-off >= 1.5.

**Overlapping upregulated pathways in CD**

| Apoptosis | Leukocyte transendothelial migration |
| --- | --- |
| Bile secretion | MAPK signaling pathway |
| Cell adhesion molecules (CAMs) | Metabolism of xenobiotics by cytochrome P450 |
| Chemokine signaling pathway | Natural killer cell mediated cytotoxicity |
| Complement and coagulation cascades | Neuroactive ligand-receptor interaction |
| Cytokine-cytokine receptor interaction | Neurotrophin signaling pathway |
| ECM-receptor interaction | NF-kappa B signaling pathway |
| ErbB signaling pathway | p53 signaling pathway |
| Estrogen signaling pathway | PI3K-Akt signaling pathway |
| Fc epsilon RI signaling pathway | Primary bile acid biosynthesis |
| Fc gamma R-mediated phagocytosis | Retinol metabolism |
| Focal adhesion | Steroid hormone biosynthesis |
| Glutathione metabolism | Thyroid hormone signaling pathway |
| Glycosaminoglycan degradation | Toll-like receptor signaling pathway |
| GnRH signaling pathway | Vascular smooth muscle contraction |
| HIF-1 signaling pathway | VEGF signaling pathway |
| Inflammatory mediator regulation of TRP channels | Wnt signaling pathway |
| Insulin signaling pathway |  |

## Ulcerative Colitis


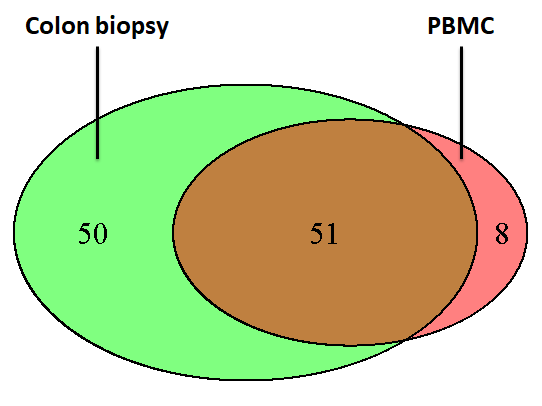


**Figure S7.4.** The venn diagram showing the overlap between up-regulated pathways in PBMCs (red) and in colon tissue (green) in ulcerative colitis. The up-regulated pathways have been filtered with PSF cut-off >= 1.5.

**Overlapping upregulated pathways in UC**

| Adipocytokine signaling pathway | Insulin secretion |
| --- | --- |
| Adrenergic signaling in cardiomyocytes | Intestinal immune network for IgA production |
| Antigen processing and presentation | Leukocyte transendothelial migration |
| Apoptosis | MAPK signaling pathway |
| Arachidonic acid metabolism | Melanogenesis |
| Axon guidance | Metabolism of xenobiotics by cytochrome P450 |
| Bile secretion | Natural killer cell mediated cytotoxicity |
| Cholinergic synapse | Neuroactive ligand-receptor interaction |
| Complement and coagulation cascades | Neurotrophin signaling pathway |
| Cytokine-cytokine receptor interaction | NF-kappa B signaling pathway |
| Drug metabolism - other enzymes | NOD-like receptor signaling pathway |
| ECM-receptor interaction | Osteoclast differentiation |
| ErbB signaling pathway | p53 signaling pathway |
| Estrogen signaling pathway | Primary bile acid biosynthesis |
| Fc epsilon RI signaling pathway | Pyrimidine metabolism |
| Fc gamma R-mediated phagocytosis | Rap1 signaling pathway |
| Focal adhesion | Ras signaling pathway |
| Galactose metabolism | Retinol metabolism |
| Glutathione metabolism | Salivary secretion |
| Glycine, serine and threonine metabolism | Steroid hormone biosynthesis |
| Glycolysis / Gluconeogenesis | Thyroid hormone signaling pathway |
| Glycosaminoglycan degradation | Tight junction |
| GnRH signaling pathway | Toll-like receptor signaling pathway |
| HIF-1 signaling pathway | Vascular smooth muscle contraction |
| Inflammatory mediator regulation of TRP channels | VEGF signaling pathway |
|  | Wnt signaling pathway |

## Sjogren's syndrome


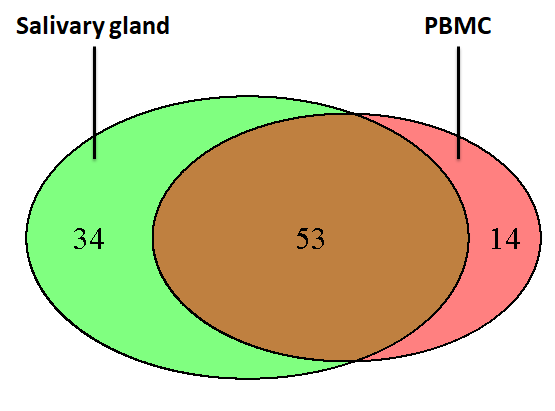


**Figure S7.5.** The venn diagram showing the overlap between up-regulated pathways in PBMCs (red) and in salivary gland (green) in Sjogren's syndrome. The up-regulated pathways have been filtered with PSF cut-off >= 1.5.

**Overlapping upregulated pathways in SS**

| Adipocytokine signaling pathway | Jak-STAT signaling pathway |
| --- | --- |
| Aminoacyl-tRNA biosynthesis | Leukocyte transendothelial migration |
| Apoptosis | MAPK signaling pathway |
| B cell receptor signaling pathway | Melanogenesis |
| Calcium signaling pathway | Mineral absorption |
| Cell adhesion molecules (CAMs) | mTOR signaling pathway |
| Cell cycle | Mucin type O-Glycan biosynthesis |
| Chemokine signaling pathway | Natural killer cell mediated cytotoxicity |
| Cholinergic synapse | Neuroactive ligand-receptor interaction |
| Circadian entrainment | Neurotrophin signaling pathway |
| Complement and coagulation cascades | NF-kappa B signaling pathway |
| Cytokine-cytokine receptor interaction | NOD-like receptor signaling pathway |
| Cytosolic DNA-sensing pathway | Osteoclast differentiation |
| Dopaminergic synapse | p53 signaling pathway |
| Endocrine and other factor-regulated calcium reabsorption | Pancreatic secretion |
| ErbB signaling pathway | PI3K-Akt signaling pathway |
| Estrogen signaling pathway | Prolactin signaling pathway |
| Fc epsilon RI signaling pathway | Rap1 signaling pathway |
| Fc gamma R-mediated phagocytosis | Ras signaling pathway |
| Focal adhesion | RIG-I-like receptor signaling pathway |
| Folate biosynthesis | Salivary secretion |
| GnRH signaling pathway | T cell receptor signaling pathway |
| HIF-1 signaling pathway | Thyroid hormone signaling pathway |
| Hippo signaling pathway | TNF signaling pathway |
| Insulin signaling pathway | Toll-like receptor signaling pathway |
| Intestinal immune network for IgA production | VEGF signaling pathway |
|  | Wnt signaling pathway |
